# Pivoting Teeth

**Published:** 1856-01

**Authors:** John Coghlan


### ARTICLE III.

*Pivoting Teeth.* By JOHN COGHLAN, M. D., M. R. C. S. Eng. &c.

"PIVOTING, though the neatest, is more frequently followed by mischievous results than any other operation performed by the dentist; and so common are these, that many surgeons consider the operation unjustifiable."—*Tomes' Den. Sur., p.* 320.

In the course of my practice as a dentist, I have had, in common with other members of the profession, frequently to regret the occurrence of inflammation, abscess and other disagreeable results of the operation of pivoting teeth. No doubt, the number of such cases may be greatly diminished, by skill and care in performing the operation; but, still, a sufficient number has hitherto remained, to have formed a standing objection to that beautiful mode of fixing artificial teeth.

The wooden pivot, so extensively and successfully used in America, is, I have reason to think, not so likely to produce alveolar abscess as the metallic one generally used in this kingdom, and throughout Europe. The difference in this respect, appears to me to depend on the wooden pivot being generally shorter, and rather slack, when pressed into the canal of the stump; the operator, depending, in a considerable degree, on the swelling of the wood to give it the necessary security. On the other hand, when a metallic pivot is used, it more nearly fills the canal, and is totally dependent for its stability on the firmness with which it is pressed in at the time. It might then, be asked, why not use the wooden pivot. In reply, I would state, that I have seen teeth pivoted with gold, still firm and perfect after having been in the mouth for 10 or 20 years! and that could not possibly be the case with wood! And again, should the canal of the stump not correspond in duration with that of the teeth to be put on, it complicated the operation considerably, and if, as sometimes occurs, the deviation be very great, it would, in my mind, form an insuperable objection to the wooden pivot.

In treating of pivoting teeth, the stumps operated upon, may most usefully be divided into two classes. In one, we may have the walls of the stump sound, but the nerve and vessels are so far destroyed, that the operation of pivoting, though with a metallic pivot, is got through without giving the slightest pain; the patient goes off much pleased, but unfortunately, his pleasure may be of short duration, as the practical dentist well knows, that those are the cases in which alveolar abscess is most likely to occur. In the other class of cases, we have the stump not only sound, but the nerve and vessels in a perfect state of integrity, so far as they are concerned; and if we except the momentary pain of destroying the nerve by the turn of a broach, we have no pain in pivoting, till we try to push our solid metallic pivot into its place, and in a large number of cases even that is got over pretty well, in consequence of the slackness of the pivot. But if in our anxiety to make a good and permanent operation, and to exclude the fluids of the mouth from the sides of the canal of the stump, we should be tempted to use a tight pivot—wriggle it up carefully as we may, still, when it gets nearly home, the patient screams out with sudden pain; and though it quickly subsides, yet we have generally, in those cases, inflammation followed by alveolar abscess.

In reasoning on the cause of these results, I came to the conclusion, that in the first case, we plug up an old secreting surface at the top of the stump, at the very time that it is probably excited into greater activity by the disturbance of the parts from the operation. In the other class, we convert the canal of the stump into a small forcing pump, and inject the column of air contained in it, by means of the tight pivot, into the sensitive tissues at the apex of the tooth. The result is inflammation, if slight it may subside and all go well; but if of a more severe character, we have secretion and consequent abscess.

Impressed as I have been for some years past with the correctness of these views, I have cut grooves in the pivot, and in fact tried every device I could think of, or that had been suggested to me, for the purpose of overcoming the difficulty; but I failed in any good *practical* result, till I thought of substituting a capillary tube for the solid pivot. The effect of the tube has been most satisfactory. By using a little care it may be used with all sorts of teeth having a thorough outlet. It may be tapped for screwing through natural teeth, or fastened by the same means as the solid pivot to the different manufactured ones. When using solder, I have sometimes found it of advantage, to suck up a little finely powdered pure plumbago and water through the minute tube of the wire, in order to prevent its being encroached upon; I have thus entirely got over the disadvantages and lost none of the advantages of the solid metallic pivot.

After having thoroughly tested the practical value of this improvement, I took a conditional protection from the patent office for this kingdom, not with the intention of securing to myself the exclusive use of it, but for the purpose of having a public record of my having originated and worked out the idea. Influenced by these feelings I give an unlimitted license to use it, to the profession at large, and the following is a copy of my specification.

"I, John Coghlan, of Wexford, in the county of Wexford, Ireland, M. D., member of the Royal College of Surgeons of England, do hereby declare, that my invention of an improved method of pivoting artificial teeth, consists in the use of a capillary tube, in lieu of the solid wire now used for that purpose.

"The advantage gained by my invention is, that the column of air necessarily contained in the canal of the prepared stump of the tooth, has an easy means of escape, and is not injected into the living tissues, as is so often the case, in using the solid pivot.

"With this capillary tube, a tooth may be at once pivoted tightly, without any disturbance of the parts; and consequently, with an enormously diminished risk of alveolar abscess.

"This method applies to the pivoting of all artificial teeth, and is perfectly new; and, in the case of old secreting stumps, it affords a ready mode of giving exit to the secretion.

"The capillary tube may be so small as not appreciably to diminish the strength of the wire, or interfere with binding it, in adopting the tusk to its proper position.

"Signed, JOHN COGHLAN.

"*August* 15*th*, 1855."

In conclusion, I would confidently express my conviction, that the use of the tube, will remove from the operation of pivoting, the odium which has hitherto been attached to it.

WEXFORD, IRELAND, *October* 13*th*, 1855.

---

